# CXCL12 chemokine and GABA neurotransmitter systems crosstalk and their putative roles

**DOI:** 10.3389/fncel.2014.00115

**Published:** 2014-04-28

**Authors:** Alice Guyon

**Affiliations:** CNRS, Institut de Pharmacologie Moléculaire et Cellulaire, UMR 7275, Université Nice Sophia AntipolisValbonne, France

**Keywords:** CXCL12/SDF1 chemokine, CXCR4, CXCR7, GABA, GABAA receptors

## Abstract

Since CXCL12 and its receptors, CXCR4 and CXCR7, have been found in the brain, the role of this chemokine has been expanded from chemoattractant in the immune system to neuromodulatory in the brain. Several pieces of evidence suggest that this chemokine system could crosstalk with the GABAergic system, known to be the main inhibitory neurotransmitter system in the brain. Indeed, GABA and CXCL12 as well as their receptors are colocalized in many cell types including neurons and there are several examples in which these two systems interact. Several mechanisms can be proposed to explain how these systems interact, including receptor–receptor interactions, crosstalk at the level of second messenger cascades, or direct pharmacological interactions, as GABA and GABA_B_ receptor agonists/antagonists have been shown to be allosteric modulators of CXCR4. The interplay between CXCL12/CXCR4-CXCR7 and GABA/GABA_A_-GABA_B_ receptors systems could have many physiological implications in neurotransmission, cancer and inflammation. In addition, the GABA_B_ agonist baclofen is currently used in medicine to treat *spasticity* in patients with spinal cord injury, cerebral palsy, traumatic brain injury, multiple sclerosis, and other disorders. More recently it has also been used in the treatment of alcohol dependence and withdrawal. The allosteric effects of this agent on CXCR4 could contribute to these beneficial effects or at the opposite, to its side effects.

## INTRODUCTION

The chemokine CXCL12/SDF1 has been found to play important roles in several processes involved in ischemic stroke and its’ subsequent repair (Wang et al., 2012), brain tumor pathogenesis (Rempel et al., 2000; Duda et al., 2011), human immunodeficiency virus (HIV) encephalopathy (Li and Ransohoff, 2008), Multiple Sclerosis and stem cell migration (Carbajal et al., 2010). This chemokine of 67 amino-acids was first believed to act on a single receptor, the CXCR4. Since then, a second receptor has been found to be another target of CXCL12, namely CXCR7 (Schonemeier et al., 2008).

CXCR4 is a G protein-coupled receptor (GPCR) widely expressed in a variety of cell types including leucocytes, where it promotes migration, recruitment and activation (Bonavia et al., 2003; Salcedo and Oppenheim, 2003; Juarez et al., 2004; Choi and An, 2011; Comerford and McColl, 2011), neurons, where it modulates electrical activity (Banisadr et al., 2002; Guyon and Nahon, 2007; Rostene et al., 2011), and various cancers and metastases (Wang et al., 2006) where it is involved in tumor progression (Liu et al., 2006; Gao et al., 2010; Zhao et al., 2010). CXCR4 also binds the HIV-1 viral envelope glycoprotein gp120 (Doranz et al., 1997; Gabuzda and Wang, 2000). Thus CXCR4 is an important therapeutic target for stroke, inflammation, neuromodulation, cancer, and in the prevention of HIV infection. CXCR4 couples to the G_i_ family of proteins activating multiple G-protein dependent pathways (Lazarini et al., 2003; Busillo and Benovic, 2007). In neurons, CXCR4 stimulation has been shown to activate a G-protein-coupled inward rectifier K^+^ (GIRK), a voltage-gated K channel Kv2.1 associated to neuronal survival, and to increase high voltage activated (HVA) Ca^2^^+^ currents (Guyon and Nahon, 2007; Shepherd et al., 2012).

CXCR7, contrary to CXCR4, could not be demonstrated to be coupled to G proteins. Despite its phylogenic relation and ligand binding properties, CXCR7 does not mediate typical chemokine receptor responses such as leukocyte trafficking. It was first believed to be mainly involved in ligand sequestration (Thelen and Thelen, 2008). However, recent studies show that ligand binding to CXCR7 activates MAP kinases through Beta-arrestins (Zabel et al., 2009; Rajagopal et al., 2010), and its functions could include modulation of circadian glucocorticoid oscillation and emotional behavior (Ikeda et al., 2013).

γ-aminobutyric acid (GABA) is the chief neuro-inhibitory neurotransmitter in mammalian systems but it also plays important roles in CNS development by regulating neurogenesis and synaptogenesis (LoTurco et al., 1995; Somogyi et al., 1995). In contrast to its inhibitory actions on adult neurons, GABA is capable of depolarizing neuronal progenitor cells and immature neurons (Ben-Ari, 2002; Rheims et al., 2008) and participates in the formation of a primitive network-driven pattern of electrical activity called giant depolarizing potentials (GDPs), which are critical for the generation of large oscillations of intracellular calcium, for activity-dependent modulation of neuronal growth and synapse formation (Ben-Ari, 2002). HIV-1 gp120, which binds and stimulates CXCR4, enhances GDPs in neonatal rat hippocampus (Kasyanov et al., 2006), underlying the role played by CXCR4 in the developmental process. Moreover, the developmental function of GABA is in part regulated by GABA production, a process mediated by glutamic acid decarboxylases (GADs), the key rate-limiting enzymes for synthesis of GABA. Two GAD isoforms, GAD65, and GAD67, are expressed in the adult nervous system (Erlander et al., 1991). It has been shown that CXCL12/CXCR4 signaling induces expression of GAD67 in embryonic hippocampal cultured neurons via ERKs and the transcription factor Egr1, a mechanism which may promote the maturation of GABAergic neurons during development (Luo et al., 2008).

The GABA type A (GABA_A_) receptors are ionotropic receptors. In response to binding GABA, their chloride-selective pore open resulting in hyperpolarization of the neuron. This causes an inhibitory effect on neurotransmission by diminishing the chance of a successful action potential occurring. The protein contains a number of different allosteric binding sites which modulate the activity of the receptor indirectly and are the targets of various other drugs, including the benzodiazepines, barbiturates, ethanol, neuroactive steroids, inhaled anesthetics, and picrotoxin, among others (Olsen and Sieghart, 2009). GABA_A_ receptors are largely expressed in the nervous system but to a limited extent they can be found in non-neuronal tissues (Mohler et al., 1995).

Like chemokine receptors, GABA_B_ receptors are GPCRs. GABA_B_ receptors are obligatory heterodimers with 2 homologous subunits (GB_1_ and GB_2_) required for functioning (Bowery et al., 1980), are widely expressed and distributed in the central nervous system (Kaupmann et al., 1998) where they can activate the GIRK channel, negatively modulate HVA Ca^2^^+^ channels and activate diverse intracellular pathways (Guyon and Leresche, 1995; Laviv et al., 2011). GABA_B_ receptors are also expressed on cells of the immune system with a possible link to the inflammatory response (Tian et al., 2004; Rane et al., 2005). As a consequence, there is a rich pharmacology aimed at targeting GABA_B_ receptors, with numerous compounds currently being used with the presumption that they are highly selective for these receptors (Bowery, 1993; Froestl, 2010).

## CO-LOCALIZATION OF CXCL12/CXCR4-CXCR7 AND GABA/GABA RECEPTOR SYSTEMS

In the periphery, CXCR4 and GABA receptors are often colocalized in the same cells. For example, in pathological conditions, CXCR4 and GABA_A_ receptors are both expressed in leukocytes (Light et al., 2013) and CXCR4 and GABA_B_ receptors are both found in cells of the immune system with a possible link to the inflammatory response (Tian et al., 2004; Rane et al., 2005; Wang et al., 2008). In the brain, CXCL12 receptors have been found to be expressed in several neuronal populations, which all express also GABA receptors (Banisadr et al., 2002; Schonemeier et al., 2008).

In the developing mouse CNS, expression of CXCR4 starts as early as embryonic day 8.5 and is sustained until adulthood. From E 15.5, both CXCR4 and CXCL12 are expressed in the cortex, olfactory bulb, hippocampus, as well as the meninges and endothelia. During postnatal development, CXCL12 influences the migration of GABAergic interneurons in the cortex by acting via CXCR4 (Stumm et al., 2003; Tiveron et al., 2006). In adults, CXCR4 immunoreactivity has been reported in many brain areas including cerebral cortex, caudate putamen, globus pallidus, substantia innominata, supraoptic, and paraventricular hypothalamic nuclei, ventromedial thalamic nucleus and substantia nigra, and in virtually all CNS cells including neurons, astrocytes, microglia, ologidendrocytes, and endothelial cells (Banisadr et al., 2002).

CXCR7, at embryonic stages, is distributed in the germinative zone of the ganglionic eminences, caudate putamen, and along the routes of GABAergic precursors migrating toward the cortex (Schonemeier et al., 2008). In the cortex, CXCR7 is expressed in GABAergic precursors and in some reelin-expressing Cajal-Retzius cells. Unlike CXCR4, CXCR7 is abundant in neurons forming the cortical plate and sparse in the developing dentate gyrus and cerebellar external germinal layer. CXCR7 is often co-localized with GAD in the postnatal cortex, hippocampus and cerebellum (Schonemeier et al., 2008). In the adult brain, CXCR7 is expressed by blood vessels, pyramidal cells in CA3, and mature dentate gyrus granule cells, which is reminiscent of the SDF-1 pattern. Further neuronal structures expressing CXCR7 include the olfactory bulb, accumbens shell, supraoptic and ventromedial hypothalamic nuclei, medial thalamus, and brain stem motor nuclei (Schonemeier et al., 2008).

Moreover, at the sub-cellular level, CXCL12 has partly a vesicular localization in axonal terminals (Reaux-Le Goazigo et al., 2012) and CXCR4 receptors are mainly located on the neuronal plasma membrane, where, like GABA receptors, they are present at pre-synaptic and post-synaptic sites of central terminals (Reaux-Le Goazigo et al., 2012).

Therefore, in the brain, the interactions between the two systems are made possible by a high level of colocalization.

## EXAMPLES OF INTERPLAY BETWEEN THE TWO SYSTEMS

CXCL12 and GABAergic agents have complementary functionality. Similarly to CXCL12, GABA, and GABAergic agents have chemotaxic properties. For example, neutrophils (Rane et al., 2005) but also stem/progenitor cells (Zangiacomi et al., 2009) and embryonic neurons (Behar et al., 1996) and are attracted by GABA. GABAergic agents have also been shown to have anti-inflammatory properties^45^. The involvement of GABA receptors has been proposed in these effects, but curiously, the putative cross-talk between the two systems has been poorly investigated.

However, several groups have described the importance of the interplay between CXCR4 and GABA_B_receptors. For example, we and others have shown inhibition of CXCL12-induced migration of cancer cells by GABA_B_ligands (Wang et al., 2008; Guyon et al., 2013). Recently, it has also been shown that CXCL12 and GABA acting on its GABA_A_ receptors interact to regulate axophilic migration of GnRH neurons (Casoni et al., 2012). GABAergic and CXCL12/CXCR4 systems interact, promoting linear rather than random movement. The simultaneous activation of these signaling pathways result in tight control of cellular speed and improved directionality along the migratory pathway of GnRH neurons (Casoni et al., 2012).

There is also evidence that CXCL12 can interact with GABA systems to modulate neurotransmission. Indeed, CXCL12 increases GABA neurotransmitter release in brain slices from different brain areas (Guyon et al., 2006; Heinisch and Kirby, 2010). Finally, agents acting on GABA receptors including GABA itself and GABA_B_ receptors agonists/antagonists have been shown to reduce the effect induced by the activation of the CXCR4 receptor on calcium currents in brain slices of substantia nigra (Guyon et al., 2013).

## PUTATIVE MECHANISMS OF INTERACTION (Figure 1)

**FIGURE 1:**
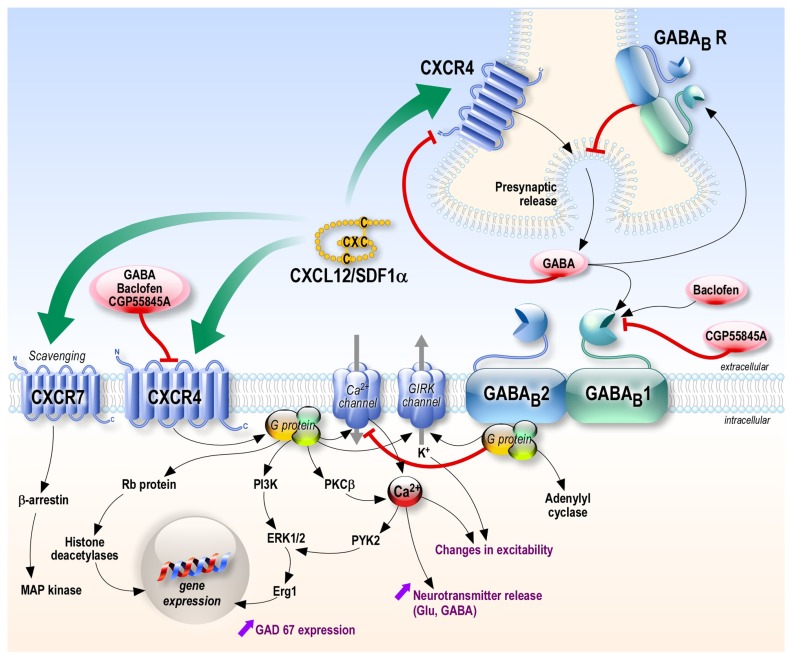
**Putative crosstalk between CXCL12-CXCR4/CXCR7 and GABAergic systems.** The chemokine CXCL12 can act on its receptors CXCR4 and CXCR7, activating several intracellular pathways. At the pre-synaptic level, CXCR4 stimulation increases the pre-synaptic release of neurotransmitter such as GABA (opposite to GABA_B_ receptors stimulation). CXCL12 receptors and GABA_B_ receptors could interact at different levels including the receptors themselves (by heteromerization), the G-proteins they activate, their common target channels (G-protein activated inward rectifier potassium channels and voltage-activated calcium channels) or the second messenger cascades. In addition, GABA is a negative allosteric modulator of CXCR4 receptors, which could contribute to the negative feedback of GABA on its presynaptic release. Black and green arrows: activation; red lines: inhibition.

### INTERACTIONS BETWEEN RECEPTORS

Although CXCR4 is also functional as a monomer (Paavola et al., 1998; Veldkamp et al., 2008, 2009), it has been shown to homo-dimerize following CXCL12 interaction, a homo-dimerization which is necessary for its functionality and signaling (Mellado et al., 2001; Toth et al., 2004), and is accompanied by receptor phosphorylation as well as changes in signal transduction processes (Rodriguez-Frade et al., 2001). This enables the activation of the JAK/STAT pathway which allows the subsequent triggering of G-protein dependent signaling events (Vila-Coro et al., 1999). CXCR4 can also form heterodimers with other GPCRs. For example, CXCR4 has been shown to form heterodimers with CXCR7, CCR2, and CCR5 and delta opioid receptors (Percherancier et al., 2005; Pello et al., 2008; Levoye et al., 2009; Sohy et al., 2009). It is therefore tempting to imagine that CXCR4 could form heterodimers with GABA_B_ receptors, which could explain the functional interactions that have been observed. However, although this has not been investigated in mammalian cell membranes, by co-expressing GABA_B_ receptors tagged with Td tomato (red fluorophore) and CXCR4 receptors tagged with GFP (green) in the membrane of *Xenopus* oocytes, data obtained using TIRF microscopy showed that CXCR4 and GABA_B_ receptors did not co-localize in the membrane (Guyon et al., 2013), thus it is unlikely that these two GPCR receptors form heterodimers.

### CROSSTALK AT THE LEVEL OF SECOND MESSENGER CASCADES

CXCR4 and GABA_B_ receptors are both GPCR activating GIRK, and modulating voltage-gated channels such as K channels Kv2.1 and HVA Ca^2^^+^ currents (Guyon and Nahon, 2007; Shepherd et al., 2012), GABA_B_ receptors stimulation decreasing HVA Ca^2^^+^ currents (Guyon and Leresche, 1995) while CXCR4 stimulation potentiating them (Guyon et al., 2008). Therefore, it is likely that the two systems might interfere at the level of the G protein, the second messenger cascade and/or the target channel in their action on neuronal excitability.

### DIRECT PHARMACOLOGICAL ACTION

While somewhat unexpected, GABA and the agonists/antagonists of GABA_B_ receptors (i.e., baclofen and the antagonists CGP55845 and 54626) were recently found to act pharmacologically directly on CXCR4 in an allosteric manner. Using electrophysiology in Xenopus oocytes and human embryonic kidney (HEK293) cells in which *Rat* CXCR4 and the GIRK channel were co-expressed, it could be demonstrated that GABA_B_ antagonist and agonist modify the CXCL12-evoked activation of GIRK channels (Guyon et al., 2013). By expressing CXCR4 receptors in heterologous systems lacking GABA_B_ receptors and performing competition binding experiments it could be investigated whether GABA_B_ ligands bind to CXCR4. Electrophysiology data and FRET experiments suggested that GABA_B_ ligands do not bind CXCR4 at the CXCL12 binding pocket suggesting allosteric modulation (Guyon et al., 2013). Finally, backscattering interferometry (BSI) on lipoparticles containing only the CXCR4 receptor allowed to quantify the CXCR4 binding affinities for the GABA_B_ ligands (including GABA), which were in a similar range to the affinities of the ligands for GABA_B_ receptors themselves, thus confirming that GABA and GABA_B_ receptor ligands directly interact allosterically with the CXCR4 receptor (Guyon et al., 2013). In the future, it will be of interest to search for putative effects of GABA and GABA_B_ receptor ligands on CXCR7.

## PHYSIOLOGICAL CONSEQUENCES

There are many pathways by which GABA and CXCL12 systems can interact. GABA is able to block the effect of CXCL12 on CXCR4. Thus, it is likely that when the GABAergic system is activated, GABA released in the brain will antagonize the effect of CXCL12 on its receptor CXCR4, and thus could influence the chemokine neurotransmission as well as the inflammatory response in the central nervous system. Conversely, it has previously been shown that CXCR4 stimulation by CXCL12 can increase GABA release (Guyon and Nahon, 2007; Bhattacharyya et al., 2008; Qu et al., 2008). Therefore, there is reciprocal cross talk between these two systems that may affect several physiological levels.

### NEUROTRANSMISSION

CXCR4 activation by CXCL12 has been shown to increase presynaptic neurotransmitter release and particularly GABA release in several neuronal populations (Guyon and Nahon, 2007; Bhattacharyya et al., 2008; Qu et al., 2008). If GABA can in turn block the effects of CXCL12, this could represent a negative feedback loop for presynaptic chemokine release (Guyon and Nahon, 2007; Bhattacharyya et al., 2008; Qu et al., 2008). Indeed, when applying CXCL12 for several minutes, a transient increase in the frequency of sPSCs is frequently observed, followed by a reduced activity (see Figure 3 in Guyon et al., 2006). This reduction could be due to an antagonistic effect of GABA, although desensitization of CXCR4 itself cannot be excluded.

In dopaminergic neurons of the rat substantia nigra, CXCR4 stimulation by CXCL12 induces an increase of release of presynaptic neurotransmitter, particularly of GABA (Guyon et al., 2006). CGP55845A (500 nM) blocks the outward GIRK current induced by CXCL12 (recorded in the presence of glutamate receptor blockers), which was first interpreted as an effect mediated through GABA_B_ receptor stimulation by GABA spilling over following CXCL12 presynaptic stimulation and increase in GABA_B_ release. However, GIRK currents might have been activated by the stimulation of postsynaptic CXCR4 by CXCL12, which was then blocked by CGP55845A.

### INFLAMMATORY RESPONSE

Expression of GABA_B_ receptors on cells of the immune system has recently been described, as well as a possible link to the inflammatory response (Tian et al., 2004; Rane et al., 2005). Along this line, it has been shown that baclofen, a selective GABA_B_ receptor agonist, reduces chemotaxis from human mononuclear cells toward CXCL12 (Duthey et al., 2010). Given that human mononuclear cells express both GABA_B_ and CXCR4 receptors, the finding that an agonist of one receptor alters the response to an agonist of the other receptor was interpreted to indicate a heterologous desensitization between chemokine and GABA_B_ receptors. This observation along with our own observations on the chemotaxis of cancer cell lines expressing CXCR4 can also be reinterpreted as a direct allosteric action of baclofen on CXCR4.

### PUTATIVE APPLICATIONS IN CANCER TREATMENT

Baclofen treatment was demonstrated to reduce the incidence of some carcinogen-induced gastrointestinal cancers in rats (Tatsuta et al., 1990) as well as human hepatocarcinoma cell growth (Wang et al., 2008). By contrast, baclofen promotes human prostate cancer cell migration (Azuma et al., 2003).

Similarly, it has been shown that GABA can affect the cell proliferation and have anti-inflammatory properties through inhibition of fibroblast proliferation, although the mechanism of action of GABA was not elucidated (Han et al., 2007). We suggest that GABA could have acted through the CXCR4 receptor, as CXCR4 is expressed on fibroblasts (Qu et al., 2008).

### HIV INFECTION

Fusion of HIV-1 with the host cell membrane is initiated by the binding of the viral envelope glycoprotein gp120 to both the CD4 cell surface receptor and one of the CXCR4 or CCR5 chemokine receptors (Doranz et al., 1997; Gabuzda and Wang, 2000). It has therefore been suggested that the CXCR4-CXCL12 axis may be an important therapeutic target for prevention of HIV infection. It will therefore be of interest to test the aptitude of baclofen and other GABA_B_ receptor agents to affect the CXCR4–GP-120 interaction.

As a conclusion, agents interacting at CXCR4 could be useful to treat cancer as well as HIV infection. Baclofen is currently approved for the treatment of *spasticity* in patients with spinal cord injury, cerebral palsy, traumatic brain injury, multiple sclerosis and other disorders (Plassat et al., 2004; Guglani and Lodha, 2007; Kolaski and Logan, 2008; Rekand and Gronning, 2011). Recently, it has been used in the treatment of alcohol dependence and withdrawal (Addolorato et al., 2006). The allosteric effects of baclofen on CXCR4 could contribute to its beneficial effects as CXCR4 often co-localizes with GABA_B_ receptors. At the opposite, it could be responsible for its side effects. Overall, the effect of GABAergic agents on CXCR4 suggests new therapeutic potentials for neurological and immune diseases.

## Conflict of Interest Statement

The author declares that the research was conducted in the absence of any commercial or financial relationships that could be construed as a potential conflict of interest.
